# Rare and localized events stabilize microbial community composition and patterns of spatial self-organization in a fluctuating environment

**DOI:** 10.1038/s41396-022-01189-9

**Published:** 2022-01-25

**Authors:** Davide Ciccarese, Gabriele Micali, Benedict Borer, Chujin Ruan, Dani Or, David R. Johnson

**Affiliations:** 1 Department of Environmental Microbiology, Swiss Federal Institute of Aquatic Science and Technology (Eawag), 8600 Dübendorf, Switzerland; 2 Department of Environmental Systems Science, ETH Zürich, 8092 Zürich, Switzerland; 3 Department of Earth, Atmospheric and Planetary Sciences, Massachusetts Institute of Technology, Cambridge, MA 02139 USA; 4 College of Land Science and Technology, China Agricultural University, 100193 Beijing, China; 5 Division of Hydrologic Sciences, Desert Research Institute, Reno, NE 89512 USA

**Keywords:** Microbial ecology, Biofilms

## Abstract

Spatial self-organization is a hallmark of surface-associated microbial communities that is governed by local environmental conditions and further modified by interspecific interactions. Here, we hypothesize that spatial patterns of microbial cell-types can stabilize the composition of cross-feeding microbial communities under fluctuating environmental conditions. We tested this hypothesis by studying the growth and spatial self-organization of microbial co-cultures consisting of two metabolically interacting strains of the bacterium *Pseudomonas stutzeri*. We inoculated the co-cultures onto agar surfaces and allowed them to expand (i.e. range expansion) while fluctuating environmental conditions that alter the dependency between the two strains. We alternated between anoxic conditions that induce a mutualistic interaction and oxic conditions that induce a competitive interaction. We observed co-occurrence of both strains in rare and highly localized clusters (referred to as “spatial jackpot events”) that persist during environmental fluctuations. To resolve the underlying mechanisms for the emergence of spatial jackpot events, we used a mechanistic agent-based mathematical model that resolves growth and dispersal at the scale relevant to individual cells. While co-culture composition varied with the strength of the mutualistic interaction and across environmental fluctuations, the model provides insights into the formation of spatially resolved substrate landscapes with localized niches that support the co-occurrence of the two strains and secure co-culture function. This study highlights that in addition to spatial patterns that emerge in response to environmental fluctuations, localized spatial jackpot events ensure persistence of strains across dynamic conditions.

## Introduction

Microbial communities frequently experience perturbations and spatiotemporal fluctuations in their local environmental conditions [1–6]. Such perturbations and fluctuations can have important effects on community stability [7] and can modulate inter- and intra-specific cell–cell interactions [8]. For example, many soil environments experience alternating cycles of wet and dry conditions that can induce changes in community composition by promoting growth during hydrated conditions [9, 10] and reducing distances between individual cells that facilitate cell–cell interactions during unsaturated conditions [11]. In coastal environments, tidal dynamics can modify environmental conditions and consequently impose changes on community composition [12] and metabolic activity [13]. On plant leaf surfaces, diurnal fluctuations can modulate resource availability and change the set of available carbon resources, which can again impose changes on community composition and metabolic activity [14]. Finally, in the human gut, changes in dietary conditions can induce changes in the structure and functioning of the gut microbiome [1]. Thus, environmental perturbations and fluctuations significantly influence the ecological and evolutionary processes governing community structure and functioning [6, 15].

One mechanism by which changes in environmental conditions can affect community structure and functioning is by modulating the types of interactions that occur between different cell-types (e.g., mutualism, commensalism, antagonism, competition, etc.) [8, 16, 17]. In turn, changes in the type of interaction can change how those cell-types arrange themselves across space (referred to hereafter as spatial self-organization) [18–21]. Importantly, spatial self-organization is a determinant of many community-level properties and behaviors [22–27], including the metabolic processes performed by microbial communities [8, 28, 29], the resistance and/or resilience of microbial communities to invasion [30, 31] and the evolutionary processes acting on microbial communities [32–36].

In populations growing in an unstructured and steady-state environment, the emergence of rare stochastic events, such as the accumulation of beneficial mutations, are referred to as jackpot events [37]. However, in spatially structured populations, the persistence of such a mutation during range expansion requires that the mutation emerge in a favorable spatial position that secures its presence at the expansion edge [38]. This is particularly relevant for sessile growth of microbial colonies, where small populations expand into adjacent unoccupied space and growth is confined to a thin layer of cells at the expansion edge [32]. In the absence of environmental perturbations, microbial communities undergoing range expansion show a decrease in diversity with only a few lineages persisting at the expansion edge [39–41]. Laboratory and in silico experiments demonstrated that stochastic processes [32, 36] and mechanical forces acting between cells [42, 43] in combination with initial spatial positioning [24, 36] can control the dynamics of diversity loss during sessile microbial range expansion. Further investigations demonstrated the importance of initial spatial positioning when sustained by the local substrate landscape, thus leading to the establishment of successful lineages at the expansion edge [44]. In other words, the presence of a specific cell-type at the expansion edge may result from stochastic processes that do not require beneficial mutations. We use the term “spatial jackpot events” to emphasize the importance of favorable initial spatial positioning [38] to position cell-types at the expansion edge while the metabolic strength guarantees their stable position at the expansion edge during environmental perturbations. Although spatial self-organization during range expansion has been frequently studied under steady-state conditions (e.g., stable redox conditions), further attention is required to understand how environmental perturbations and fluctuations affect microbial interactions and spatial self-organization.

In this study, we investigated the stability of a cross-feeding microbial co-culture under fluctuating environmental conditions. We hypothesized that temporal fluctuations in environmental conditions that alter the nature of interspecific interactions can lead to irreversible transitions in spatial patterns of cell-types, thus affecting co-culture composition and metabolic functioning. Our hypothesis is based on the following two assumptions: (1) environmental conditions that foster different types of interspecific interactions promote the formation of different patterns of spatial self-organization, and (2) the patterns of spatial self-organization that emerge under one set of environmental conditions can alter co-culture composition, spatial self-organization, and functioning under a different set of environmental conditions. The above assumptions are not met if spatial jackpot events emerge that enable cell-types to maintain a stable position at the expansion edge.

**Fig. 1:**
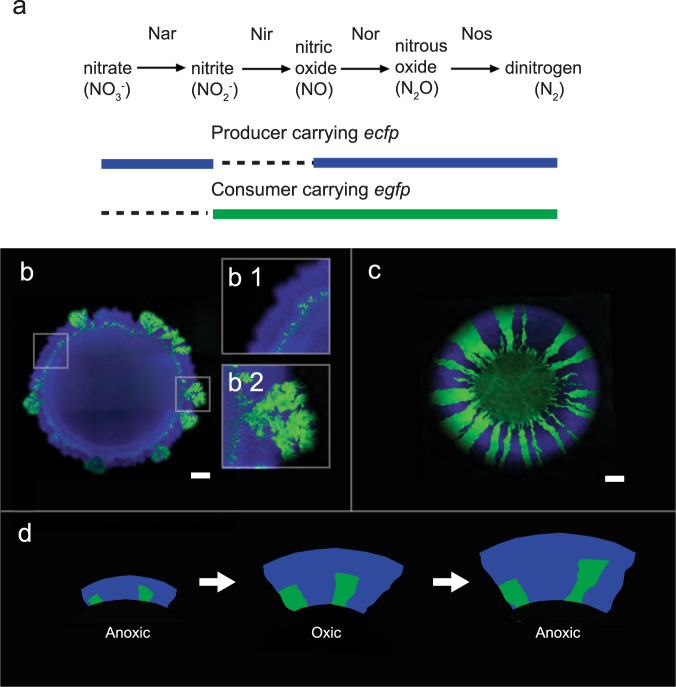
Two-strain microbial co-culture used in this study. **a** The co-culture is composed of two isogenic mutant strains of *P. stutzeri* that differ in their ability to reduce nitrate (NO_3_^−^) and nitrite (NO_2_^−^). One strain can reduce nitrate but not nitrite (referred to as the producer; solid blue horizontal lines) whereas the other can reduce nitrite but not nitrate (referred to as the consumer; solid green horizontal line). The two strains also carry either the *ecfp* blue or *egfp* green fluorescent protein-encoding gene. Different patterns of spatial self-organization emerge depending on redox conditions. **b**1 Anoxic conditions induce a mutualistic interaction and “producer-first expansion”, where the producer expands ahead of the consumer. This is because the consumer cannot grow until the producer begins producing nitrite. **b**2 The community is punctuated by individual “consumer-first expansion” patterns that persist to the expansion edge (referred to as spatial jackpot events). **c** Oxic conditions induce a competitive interaction and “simultaneous expansion” of the two strains, resulting in segregated sectors with interspecific boundaries lying approximately parallel to the expansion direction. The scale bars are 1000 μm. **d** In a fluctuating environment, the previous range expansion determines the initial spatial positionings of the strains for the subsequent range expansion, and may thus fundamentally alter spatial self-organization. We predict that repeated transitions between anoxic and oxic conditions will result in a gradual decrease in the ratio of consumer-to-producer, thus potentially leading to the accumulation of nitrite to toxic concentrations. This is due to the preferential spatial positioning of the producer at the onset of oxic conditions.

We tested this hypothesis with a microbial co-culture that satisfies both of the above-mentioned assumptions. The component strains engage in competition under oxic conditions and mutualistic cross-feeding of the conditionally toxic metabolite nitrite (NO_2_^−^) under anoxic conditions (Fig. 1a). Oxic and anoxic conditions promote the formation of fundamentally different patterns of spatial self-organization [19] (Fig. 1b, c), satisfying the first assumption discussed above. In addition, we predict that the patterns of spatial self-organization that emerge under oxic conditions are detrimental to the co-culture as a whole under anoxic conditions, satisfying the second assumption discussed above (Fig. 1d). Briefly, anoxic conditions result in a dominance of the nitrite-producing strain (referred to as the producer) at the expansion edge [19, 34, 45] (Fig. 1d). If the environment changes to oxic conditions, the producer will have preferential access to resources supplied via diffusion from the periphery and will increase in abundance relative to the nitrite-consuming strain (referred to as the consumer). If the environment switches back to anoxic conditions, the increased relative abundance of the producer will result in nitrite accumulation. Over a series of anoxic/oxic transitions, we predict a continual increase in the relative abundance of the producer and the potential accumulation of nitrite to toxic concentrations, thus creating detrimental conditions for the co-culture as a whole. We tested this prediction by repeatedly transitioning the environment between anoxic and oxic conditions and quantifying the effects on co-culture composition and local spatial organization at the expansion edge.

## Materials and methods

### Experimental system

The experimental microbial co-culture consists of two isogenic mutant strains of the bacterium *Pseudomonas stutzeri* A1501 [19, 34, 46, 47] (Fig. 1a). The producer has a single loss-of-function deletion in the *nirS* gene and can reduce nitrate (NO_3_^−^) to nitrite (NO_2_^−^) but not nitrite to nitric oxide (NO) (Fig.1a). The consumer has a single loss-of-function deletion in the *narG* gene and cannot reduce nitrate to nitrite but can reduce nitrite to nitric oxide (Fig. 1a). The two strains differ at only single genetic loci [46], thus preventing potential confounding effects that might otherwise occur if more distantly related strains were used. Both strains have an intact periplasmic nitrate reductase encoded by the *nap* genes; however, this reductase does not support growth with nitrate under anoxic conditions [46, 48] and is likely involved with the dissipation of excess reducing equivalents rather than respiration [48]. To avoid recombination between the two strains when grown together, both have a single loss-of-function deletion in the *comA* gene [46]. Finally, to distinguish the two strains when grown together, each has a chromosomally-located *ecfp* or *egfp* fluorescent protein-encoding gene, which encode for cyan and green fluorescent protein respectively (Fig. 1) [34, 46, 47].

We imposed different interactions between the producer and consumer by controlling the redox conditions of the environment. When the strains are grown together under anoxic conditions with nitrate (NO_3_^−^) as the growth-limiting resource, they engage in a mutualistic nitrite (NO_2_^−^) cross-feeding interaction [46]. Nitrite is toxic at low pH, and we can therefore control the strength of the mutualistic interaction between strong and weak by setting the pH of the growth medium to 6.5 or 7.5, respectively. We validated the mutualistic interaction by demonstrating that the biomass of the producer increases when in the presence of the consumer at pH 6.5 (two-sample two-sided *t*-test*; p* = 1.3 × 10^−7^, *n* = 5) (Supplementary Fig. S1). pH itself has no quantifiable effects across this pH range, and there are no experimentally observable effects of nitrite toxicity at pH 7.5 [45, 46]. When the strains are grown together under oxic conditions, they engage in a competitive interaction for nutrients, oxygen and space [19]. For simplicity, we will henceforth only use the terminology oxic and anoxic (implying conditions that induce competitive and mutualistic interactions, respectively) and pH 6.5 and 7.5 (implying conditions that induce strong and weak mutualistic interactions, respectively).

### Range expansion experiments and temporal fluctuations

We performed range expansion experiments where we inoculated the surface of a lysogeny broth (LB) agar plate with mixtures of the producer and consumer and allowed the co-cultures to grow and expand across space [34]. To accomplish this, we first grew the producer and consumer alone with oxic LB medium overnight in a shaking incubator at 37 °C and 220 rpm. After reaching stationary phase, we centrifuged the cultures at 5488 × *g* for 8 min at room temperature, discarded the supernatants, suspended the remaining cells in 1 ml of saline solution (0.89% NaCl, w/w) and adjusted the densities of the producer and consumer independently to an optical density of one at 600 nm (OD_600_). We then mixed the producer and consumer at a volumetric ratio of 1:1 (producer to consumer) and deposited 1 µl of each mixture onto the center of a separate LB agar plate containing 1 mM of sodium nitrate (NaNO_3_). Because the producer and consumer are isogenic mutants with identical optical properties, equivalent OD_600_ values correspond to approximately equivalent cell numbers. Prior to inoculating the LB agar plates, we adjusted the pH of the molten agar to 6.5 or 7.5 as described elsewhere [34].

We imposed transitions between anoxic and oxic conditions for fifteen cycles (*n* = 4 for each pH condition). We imposed anoxic conditions by placing the LB agar plates inside a glove box (Coy Laboratory Products, Grass Lake, USA) containing a nitrogen (N_2_):hydrogen (H_2_) atmosphere (97:3) and oxic conditions by placing the LB agar plates in ambient air. We performed oxygen measurements and confirmed that all available oxygen had diffused out of the LB agar plate within 12 h of transferring the plates back into the glove box (Supplementary Fig. S2). A single cycle consisted of incubation for 36 h under anoxic conditions followed by 12 h under oxic conditions. This provided the strains with approximately equivalent expansion opportunity under anoxic and oxic conditions. More specifically, the anoxic growth rates of the two strains with nitrate (NO_3_^−^) or nitrite (NO_2_^−^) are approximately three-fold slower when compared to their oxic growth rates [46], and we therefore provided approximately three-fold more time to expand under anoxic conditions than under oxic conditions.

### Microscopy and image acquisition

We obtained tile scans of the range expansions with a Leica TCS SP5 II confocal laser-scanning microscope (CLSM) (Leica Microsystems, Wetzlar, Germany) with a 5x HCX FL air immersion lens, a numerical aperture of 0.12, a frame size of 1024 × 1024, and a pixel size of 3.027 µm [19, 34, 45]. We set the laser emission to 458 nm for the excitation of cyan fluorescent protein (encoded by the *ecfp* gene) with an emission range of 480–493 nm and to 488 nm for the excitation of green fluorescent protein (encoded by the *egfp* gene) with an emission range of 510–559 nm [19, 34, 45]. We scanned the range expansions at every transition between anoxic and oxic conditions. We set the illumination plane to capture the expansion edge where active expansion and self-organization was ongoing rather than the shallower and non-expanding center. We exposed the agar plates to ambient air for 1 h prior to image acquisition to allow for maturation of the fluorescent proteins [34].

### Quantitative image analysis

We processed the CLSM images using ImageJ (imagej.net) and MATLAB R2017a (MathWorks, Nantick, MA, USA). We provide a detailed description of the image processing method used in this study in the Supplementary Text and Supplementary Fig. S3. We quantified co-culture composition and local spatial arrangement at the expansion edge using two measurements; the ratio of consumer-to-producer (indicating the relative abundance of the two strains) and the intermixing index (a measure of local spatial organization). We quantified the ratio of consumer-to-producer within a ring located at the expansion edge using a circular windowing approach, where the outer edge of the ring was located at the expansion edge and the inner edge of the ring was located 50 pixels (which correspond to 151.35 µm) behind the expansion edge (Supplementary Fig. S3). We selected this area to avoid overlap between the focal and previous time points, thus ensuring that time-consecutive measures of the ratio of consumer-to-producer were non-overlapping. We validated our measurements of the ratio of consumer-to-producer by comparing them to those obtained with conventional colony forming unit plate counting (Supplementary Fig. S4). We quantified the intermixing index, which measures the degree of spatial intermixing between the two strains, within a circle located at a radial distance of 50 pixels from the expansion edge as described elsewhere [34, 49, 50] (Supplementary Fig. S3).

### Statistical analyses

We used parametric methods for all of our statistical tests and considered *p* < 0.05 to be statistically significant. We used the Wilk–Shapiro test to test for normality and the Bartlett test to test for homoscedasticity of our datasets and considered *p* > 0.05 to validate the assumptions of our parametric tests (i.e., we found no evidence that our datasets significantly deviate from the assumptions of normality and homoscedasticity). We reported the type of statistical test, the sample size for each test, and the exact *p* for each test in the results section. We performed all statistical test using MATLAB R2017a (MathWorks, Nantick, MA, USA).

### Agent-based mathematical model

We simulated co-culture expansion using a model that combines pseudo two-dimensional nutrient diffusion with an agent-based representation of microbial cells with localized growth conditions calculated using Monod-type kinetics. We reported details of the model including all equations elsewhere [44]. Briefly, we created a hexagonal lattice with a side length of 20 microns that we used as a backbone for diffusion calculations in a spherical domain. We used a total diameter of 1 cm based on one-dimensional Fickian diffusion between nodes while respecting mass balance at each node. We considered only carbon, nitrate (NO_3_^−^) and nitrite (NO_2_^−^) in the simulations and we assumed other nutrients are not growth-limiting. We set constant peripheral sources for carbon and nitrate at concentrations of 22 and 1 mM, respectively, where nitrite is produced solely by the metabolic activity of the producer. We represented microbial cells as super agents [51] where each grid node is inhabited by one strain (i.e., the two strains are mutually exclusive). We inoculated the domain with cells at the center (radius of 2 mm) and attributed each node randomly with either a producer or consumer cell. We randomly set the initial mass of each cell to be between 10 and 100% of the mass at division.

We calculated microbial growth rates using Monod-type kinetics. Under oxic conditions, both strains consume carbon as the growth-limiting nutrient at the expansion edge, and we therefore only added a carbon limitation term. Under anoxic conditions, we added a nitrate (NO_3_^−^) limitation term for the producer and a nitrite (NO_2_^−^) limitation term for the consumer. We updated biomass using the explicit Euler method and related nutrient consumption at each grid node to the growth rate using yield coefficients. Nitrite is produced by the producer and consumed by the consumer according to stoichiometry. Nitrite toxicity was shown to primarily influence the growth yield [52]. Thus, we used the following equation to calculate the biomass yield coefficient for each genotype:

1 $$Y_i = \left\{ {\begin{array}{*{20}{c}} {\left( {Y_{{{{{\rm{{max}}}}}}} - Y_{{{{{\rm{{min}}}}}}}} \right) \times \frac{{K_{{{{{\rm{{inh}}}}}}}}}{{K_{{{{{\rm{{inh}}}}}}} + C_{{{{{\rm{{NO2,i}}}}}}}}} + Y_{{{{{\rm{{min}}}}}}},\;{{{{{\rm{pH}}}}}} = 6.5} \\ \qquad\qquad\qquad\qquad\quad\;\qquad{Y_{{{{{\rm{{max}}}}}}},\;{{{{{\rm{pH}}}}}} = 7.5} \end{array}} \right.$$

where *Y*_max_ is the maximum biomass yield (kg dry weight/mol), *Y*_min_ is the minimum biomass yield (kg dry weight/mol), and *K*_inh_ is the nitrite inhibition coefficient (mM).

We simulated co-culture expansion through cell division combined with a mechanical cell shoving algorithm. Upon reaching a defined mass at division, microbial cells divide into two, where one daughter cell occupies the current location and the other either occupies an adjacent grid node (if the node is unoccupied or cell shoving is possible) or is aggregated at the current location in a layer above (pseudo three-dimensional colony growth). From the grid node of a dividing cell, the shortest distance to the expansion edge is calculated. If the distance is sufficiently small (<100 µm, 5 grid nodes), then all cells along the shortest path are shoved toward the edge. The current cell at the edge is then shoved to a new node at random from any of the unoccupied neighboring nodes.

We set the total simulation time to 72 h using a 60 s time-step. During this time, we alternated the environment between anoxic and oxic conditions every 6 h (i.e., a total of six intervals per condition). In comparison to the experiments, we simulated the growth rates of the strains according to oxic conditions and did not alter the parameters for anoxic conditions for sake of parameter parsimony. Congruent to the experiment, this parametrization enabled equal division during anoxic and oxic conditions. We note that the consumer does have a slightly slower growth rate than the producer under experimental anoxic conditions [34], but accounting for this has no effect on the qualitative outcomes of the simulations.

## Results

### Effects of environmental fluctuations on co-culture composition and intermixing

We first tested the effects of fluctuations between anoxic (inducing a mutualistic interaction) and oxic (inducing a competitive interaction) conditions on co-culture composition (quantified as the ratio of consumer-to-producer at the expansion edge) and interspecific mixing (quantified as the number of interspecific boundaries divided by the colony circumference). We expected that, over a series of anoxic/oxic transitions, the ratio of consumer-to-producer at the expansion edge and the degree of intermixing would both decrease (Fig. 1d). To test this, we performed range expansions where we transitioned the environment between anoxic and oxic conditions. While we performed the experiments with defined anoxic and oxic incubation times, our main prediction (i.e., that repeated transitions between anoxic and oxic conditions can induce irreversible pattern transitions that alter co-culture composition and functioning) is independent of the time spent under either of those conditions as far as cells can adjust their metabolism to the new environment (Fig. 1d).

**Fig. 2:**
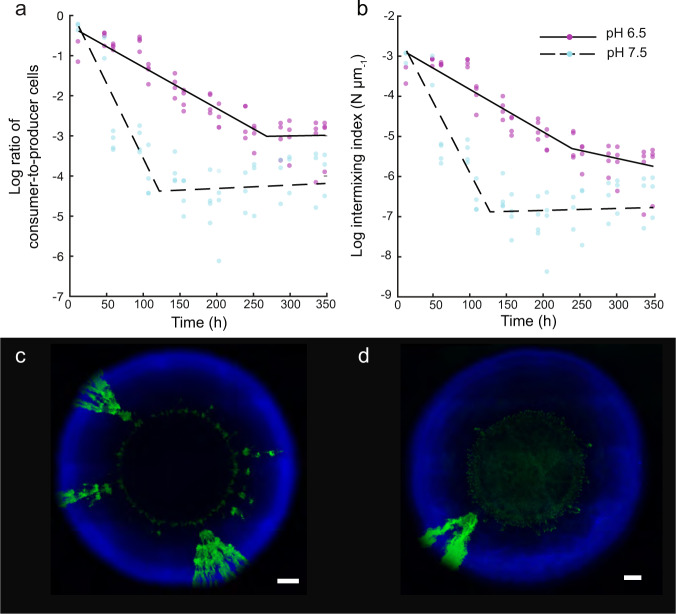
Dynamics of co-culture composition and intermixing during repeated anoxic/oxic transitions. **a** Co-culture composition measured as the ratio of consumer-to-producer. **b** Intermixing between the consumer and producer measured as the intermixing index, where *N* is the number of interspecific boundaries between the two strains. Experiments were performed at pH 6.5 (strong mutualistic interaction) (magenta data points) or pH 7.5 (weak mutualistic interaction) (cyan data points). Each data point is for an independent replicate (*n* = 4). The solid black lines are the two-phase linear regression models for pH 6.5, while the dashed black lines are the two-phase linear regression models for pH 7.5. Images of the final expansions after 350 h of incubation at **c** pH 6.5 and **d** pH 7.5. The scale bars are 1000 μm.

As expected, the ratio of consumer-to-producer and the intermixing index both decreased over the series of anoxic/oxic transitions (Fig. 2a, b). The changes in these quantities appear to have two distinct dynamic phases; a first phase with a relatively steep decay and a second phase with a shallower decay. We therefore modeled their dynamics using a two-phase linear regression model [53–55]. During the first phase, the ratio of consumer-to-producer decreased significantly more rapidly at pH 7.5 (*r*^2^ = 0.90, *p* = 2 × 10^−9^, coeff = −0.0374, 95% CI = [−0.038, −0.0368]) than at 6.5 (*r*^2^ = 0.94, *p* = 1 × 10^−7^, coeff = −0.0103, 95% CI = [−0.0108, −0.0097]) (Fig. 2a). We observed consistent results for the intermixing index, where it also decreased significantly more rapidly at pH 7.5 (*r*^2^ = 0.90, *p* = 2 × 10^−9^, coeff = −0.0289, 95% CI = [−0.0295, −0.0284]) than at 6.5 (*r*^2^ = 0.93, *p* = 9 × 10^−8^, coeff = −0.01, 95% CI = [−0.0109, −0.0098]) (Fig. 2b). During the second phase, the change in the ratio of consumer-to-producer did not significantly differ between pH 7.5 (*r*^2^ = 0.90, *p* = 2 × 10^−9^, coeff = 0.0008, 95% CI = [0.0002, 0.0014]) and 6.5 (*r*^2^ = 0.94, *p* = 1 × 10^−7^, coeff = 0.0003, 95% CI = [−0.0002, 0.0008]) (Fig. 2a). However, we observed that the decrease in the intermixing index was significantly different between pH 7.5 (*r*^2^ = 0.94, *p* = 2 × 10^−9^, coeff = 0.0018, 95% CI = [0.0013, 0.0024]) and 6.5 (*r*^2^ = 0.94, *p* = 8 × 10^−8^, coeff = −0.0019, 95% CI = [−0.0025, −0.0013]). Overall, the final ratio of consumer-to-producer is lower at pH 7.5 (mean = 0.0163, SD = 0.01) than at 6.5 (mean = 0.052, SD = 0.02) (two-sample two-sided *t*-test; *p* = 0.03, *n* = 4) (Fig. 2). Consistently, the final intermixing index is also lower at pH 7.5 (mean = 0.0039, SD = 0.0032) than at 6.5 (mean = 0.0107, SD = 0.0049) (two-sample two-sided *t*-test; *p* = 0.05, *n* = 4) (Fig. 2b).

The results described above yielded two important outcomes. First, the modeled two-phase linear regression of the ratio of consumer-to-producer and the intermixing index both depended on the strength of the mutualistic interaction, where the initial rate of decay was faster at pH 7.5 than at 6.5 (Fig. 2a, b). Thus, as the strength of the interdependency increases, the decay in the ratio and the intermixing index slows. Second, at pH 6.5 we never observed the complete loss of the consumer from the expansion edge (i.e., neither the ratio of consumer-to-producer nor the intermixing index reached zero) (Fig. 2a, b), which is counter to our initial expectation (Fig. 1d).

We further performed controls under continuous oxic and continuous anoxic conditions (Supplementary Fig. S5). The ratio of consumer-to-producer and the intermixing indices both significantly differed between continuous oxic and continuous anoxic conditions regardless of the pH (two-sample two-sided *t*-tests; *p* < 0.05, *n* = 5) (Supplementary Fig. S5). Thus, these two quantities of spatial self-organization depend on the environmental conditions. The ratio of consumer-to-producer and the intermixing indices also significantly differ between continuous oxic and fluctuating conditions, again regardless of the pH (two-sample two-sided *t*-tests; *p* < 0.05, *n*_1_ = 4; *n*_2_ = 5) (Supplementary Fig. S5). This provides evidence that these two quantities are significantly modulated by periods of anoxic conditions. However, the ratio of consumer-to-producer and the intermixing indices were not consistently significantly different between continuous anoxic and fluctuating conditions (Supplementary Fig. S5). Thus, periods of anoxic conditions appear to have larger effects on these two quantities than do periods of oxic conditions, which would be expected as anoxic conditions create an interdependency between the strains.

### The number of spatial jackpot events depend on pH

**Fig. 3:**
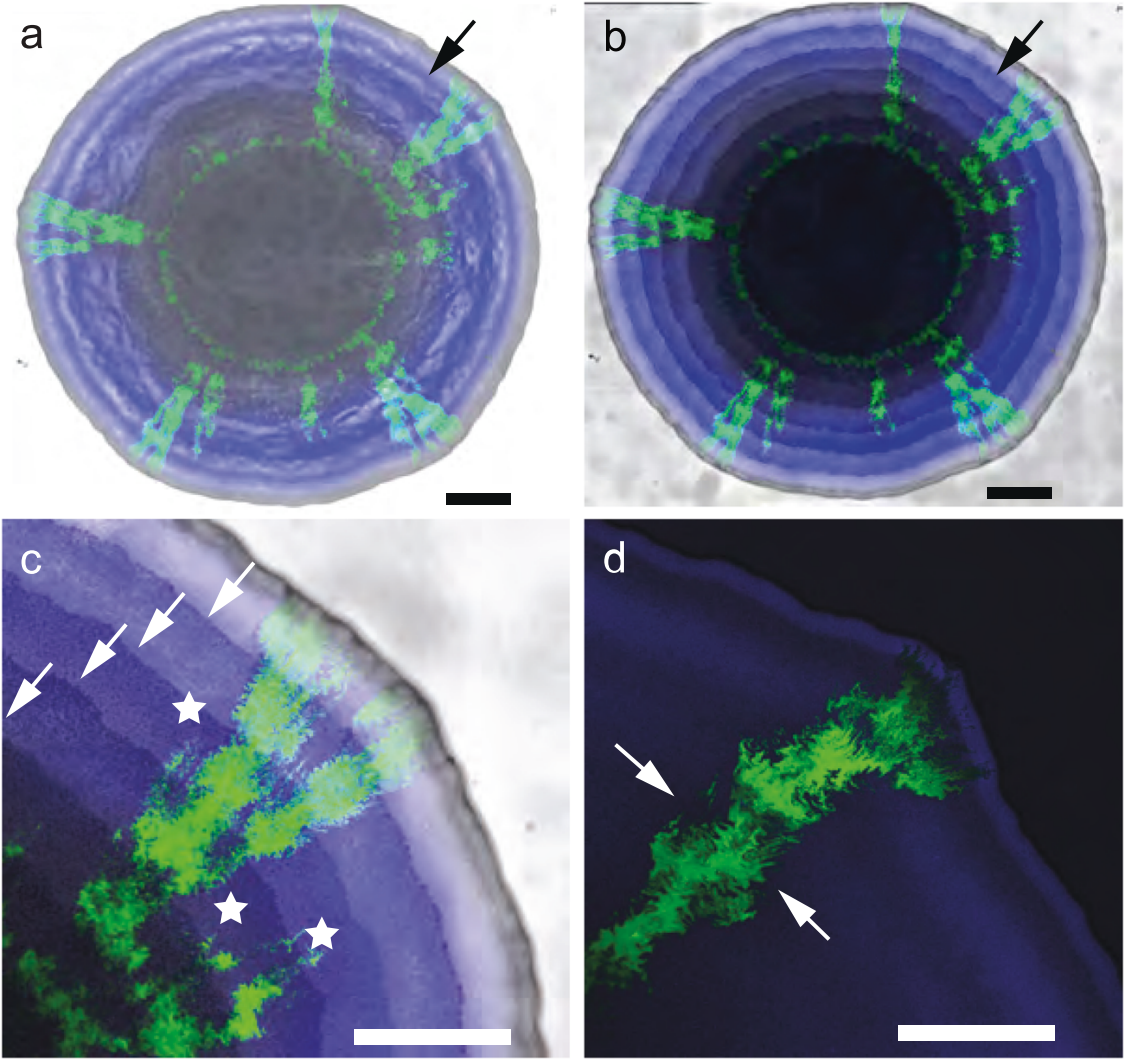
Formation and persistence of spatial jackpot events during repeated anoxic/oxic transitions. Images are after 350 h of range expansion. **a** Using reflected light, the surface morphology of the entire expansion area is visible. Transitions between anoxic (mutualistic interaction) and oxic (competitive interaction) conditions are imprinted in the expansion biomass as concentric rings (black arrow). **b** The transitions between anoxic and oxic conditions (black arrow) are more visible using the bright field. **c** Detail of the spatial jackpot events that developed during different incubation conditions. White stars indicate spatial jackpot events that did not advance to the expansion edge while the white arrows indicate transitions between anoxic and oxic conditions. **d** Transitions between anoxic and oxic conditions caused a change in the spatial self-organization of spatial jackpot events. The white arrows indicate a decrease followed by an increase in width. All scale bars are 1000 μm.

**Fig. 4:**
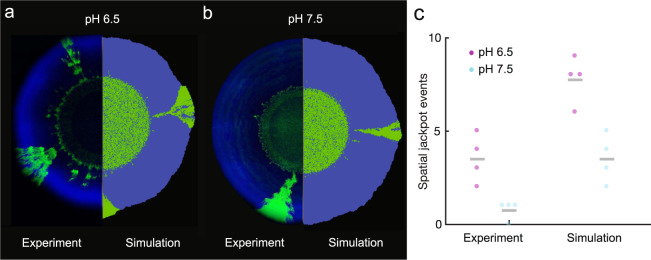
Comparison of experimental and simulated patterns of spatial self-organization. At both **a** pH 6.5 (strong mutualistic interaction) and **b** pH 7.5 (weak mutualistic interaction), the producer-first expansion pattern dominates the expansion area. However, both pH conditions foster the emergence of spatial jackpot events. **c** The cyan data points are the numbers of spatial jackpot events that persisted to the expansion edge at pH 7.5. The magenta data points are the numbers of spatial jackpot events that persisted to the expansion edge at pH 6.5. Means are indicated by the gray lines. Congruent to experimental observations, the predicted number of spatial jackpot events in the numerical simulations is higher at pH 6.5 than at 7.5.

We next tested whether the number of spatial jackpot events that emerge during range expansion depend on the pH, and thus on the strength of the mutualistic interaction. Here, we define a spatial jackpot event as a continuous region of the consumer that persists to the expansion edge. We found that the number of spatial jackpot events was higher at pH 6.5 than at 7.5 (Figs. 3 and 4). We observed mean numbers of spatial jackpot events of 3.5 (SD = 1.3, *n* = 4) at pH 6.5 and 0.75 (SD = 0.5, *n* = 4) at pH 7.5, and these mean numbers are significantly different from each other (two-sample two-sided *t*-test*; p* = 0.007, *n* = 4) (Figs. 3 and 4c). Thus, the number of spatial jackpot events is larger at pH 6.5 and slows the observed decay in the ratio of consumer-to-producer and the intermixing index over repeated transitions between anoxic/oxic conditions (Fig. 2).

### Agent-based model elucidates putative mechanisms for the persistence of spatial jackpot events

To provide further support that the number of spatial jackpot events that emerge during range expansion depends on the pH, and thus on the strength of the mutualistic interaction, we simulated range expansions under fluctuating environmental conditions using an agent-based mathematical model (Fig. 4a, b). While the experiments performed in this study reveal the spatial distributions of strains at the population level, the mathematical model captures the growth dynamics throughout the range expansion at the single-cell level and relates observed processes (such as the nucleation of spatial jackpot events and persistence during range expansion) to the underlying growth dynamics and associated substrate landscape.

**Fig. 5:**
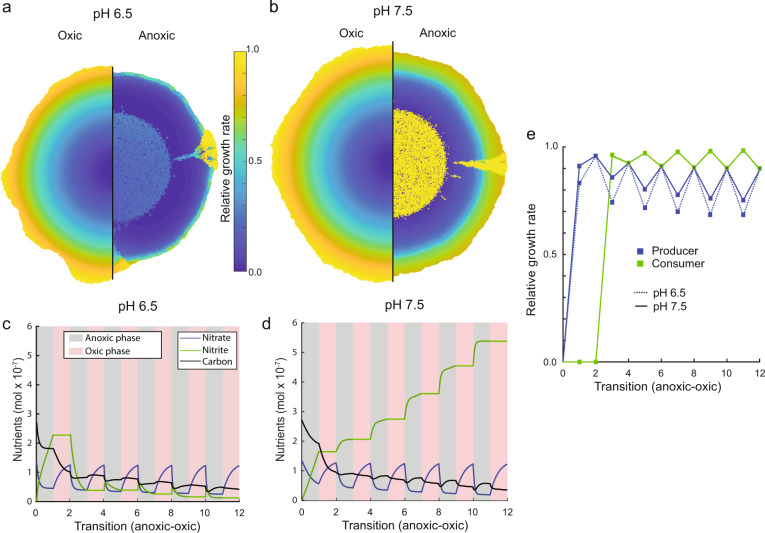
Simulations of local growth rates and nutrient dynamics during range expansion. Spatially resolved relative growth rates (realized growth rate divided by maximum growth rate) for **a** strong and **b** weak mutualistic interactions. Under oxic conditions (competitive interaction), growth rates declined radially from the periphery to the center due to carbon limitation. Under anoxic conditions (mutualistic interaction), growth rates also declined radially for the producer due to nitrate (NO_3_^−^) limitation, whereas the consumer benefitted from the ubiquitous availability of nitrite (NO_2_^−^). Total nutrient content in the simulated domain for **c** strong and **d** weak mutualistic interactions. In comparison to static anoxic conditions, nitrate limitation was less prominent due to diffusion of nitrate into the expansion area during oxic conditions. **c** For a strong mutualistic interaction, nitrite concentrations were low due to the overall higher relative abundance of the consumer. **d** For a weak mutualistic interaction, nitrite accumulated within the domain due to a lack of strong nitrite toxicity. **e** When comparing growth rates between weak and strong mutualistic interactions, the producer has a larger difference in growth rate between the two conditions whereas the consumer has a smaller difference.

We found that during anoxic conditions, nitrate (NO_3_^−^) is consumed by the producer, resulting in the formation of a nitrate gradient with low concentrations at the expansion origin and higher concentrations at the expansion edge (Fig. 5a). During oxic conditions, the producer does not consume nitrate and nitrate diffuses deep into the expansion area, which diminishes or even eliminates the previously established radial nitrate gradient (Fig. 5a, b). This reduces the effect of nitrate limitation and equilibrates the growth rates of the two strains (i.e., there is a less pronounced relative growth rate advantage of the consumer) (Fig. 5a, b). At pH 6.5, nitrite (NO_2_^−^) toxicity slows the growth of the producer and prevents nitrite from accumulating significantly (Fig. 5c). In comparison, at pH 7.5 nitrite accumulates to larger concentrations and there is a smaller relative difference in growth rates between the producer and consumer at the expansion edge (Fig. 5d).

These underlying processes affect the numbers and persistence of spatial jackpot events during fluctuations between anoxic and oxic conditions. The high relative growth rate difference between the producer and consumer at pH 6.5 fosters persistence of the consumer at the expansion edge (Fig. 5a) leading to a higher number of spatial jackpot events that protrude to the expansion edge (Fig. 4c). At pH 7.5, the absence of nitrite (NO_2_^−^) toxicity results in a less prominent growth rate difference between the producer and consumer (Fig. 5b) and thus overall lower numbers of spatial jackpot events congruent with experimental observations (Fig. 4c).

### Stability of co-culture composition and intermixing during environmental fluctuations

**Fig. 6:**
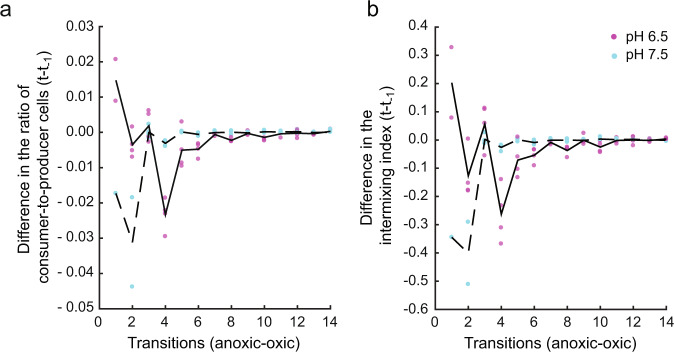
Difference in the ratio of consumer-to-producer and the intermixing index between two subsequent environmental transitions. **a** The difference in the ratio of consumer-to-producer between two subsequent transitions (anoxic/oxic) has a large variance at earlier times and reaches zero (i.e., stability) after seven transitions at pH 7.5 (weak mutualistic interaction). In contrast, the variance reaches zero after 12 transitions at pH 6.5 (strong mutualistic interaction). **b** The difference in the ratio of intermixing indices between two subsequent transitions (anoxic/oxic) reaches zero after three transitions at pH 7.5 and after 14 transitions at pH 6.5. The solid black lines are the means at pH 6.5 while the dashed black lines are the means at pH 7.5.

We next tested whether a steady-state co-culture composition and pattern of spatial self-organization emerges during repeated transitions between anoxic and oxic conditions. Here, we refer to stability as a lack of change in quantitative measures of co-culture composition and spatial self-organization over time. To test this, we quantified two spatial features; the ratio of consumer-to-producer (Fig. 6a) and the intermixing index (Fig. 6b). When tracking the two quantities over the 15 anoxic/oxic transitions, we observed that the two quantities evolve toward constant non-zero values with decreasing variance at both pH 6.5 and 7.5. The variance analysis reveals that the ratio of consumer-to-producer at pH 7.5 reaches a constant value more rapidly than at pH 6.5. The constant value emerges after seven transitions at pH 7.5 and after 12 transitions at pH 6.5. The variance in the intermixing index reaches zero after three transitions at pH 7.5 compared to the last transition at pH 6.5. This suggests that the producer is strongly dependent on the consumer when nitrite (NO_2_^−^) toxicity is high (pH 6.5), and there are likely stronger benefits for maintaining more balanced ratios of consumer-to-producer and increased intermixing (e.g., the producer advances slowly without the consumer in close spatial proximity to consume nitrite). In contrast, the variance in the ratio of consumer-to-producer and the intermixing index reaches zero earlier at pH 7.5 than at 6.5. This is intuitive, as the producer is less dependent on the consumer when nitrite toxicity is low, and there are therefore weaker benefits for maintaining balanced ratios of consumer-to-producer and intermixing (e.g., the producer can advance without the consumer).

### Effect of initial environmental conditions

**Fig. 7:**
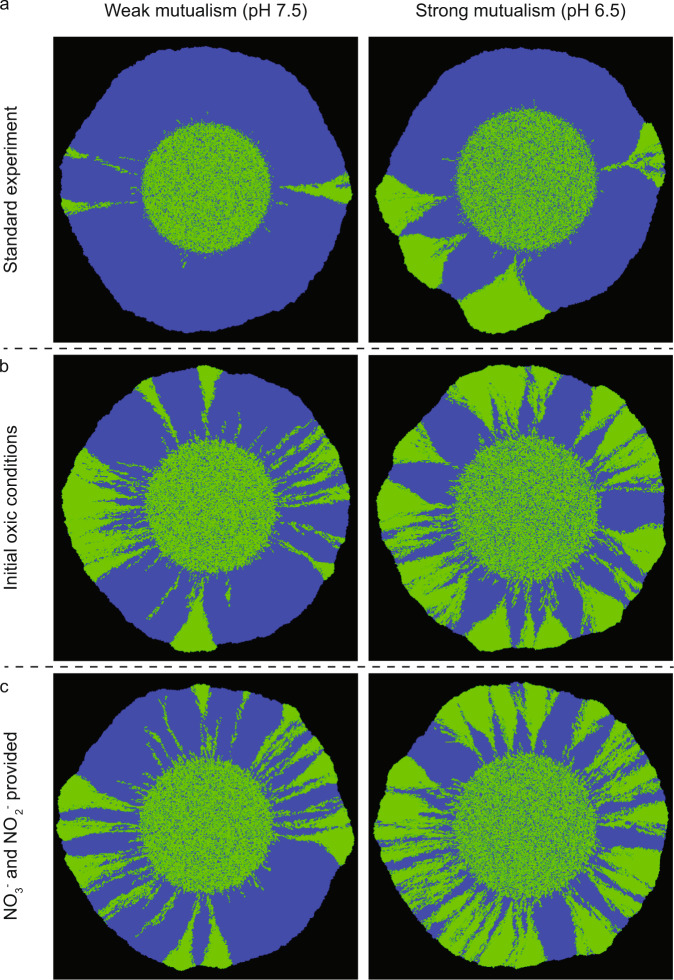
Simulations with different initial environmental conditions. **a** Standard experimental design with initially anoxic conditions and nitrate (NO_3_^−^) added exogenously as the growth-limiting substrate. **b** When expansion was initiated under oxic conditions, more spatial jackpot events emerged due to the initial growth of consumer cells at the expansion edge. **c** When expansion was initiated with an exogenous supply of both nitrate and nitrite (NO_2_^−^), the interdependence between the consumer and producer was alleviated and more spatial jackpot events proliferated to the expansion edge.

Our experiments show that the strength of the mutualistic interaction is an important determinant of the numbers and persistence of spatial jackpot events. However, this outcome could be additionally influenced by the initial environmental conditions. We thus used mathematical modeling to test how the initial environmental conditions shape the final patterns of spatial self-organization by varying the initial redox conditions as well as the availability of growth-limiting nutrients (i.e., by providing nitrite [NO_2_^−^] in addition to nitrate [NO_3_^−^]) (Fig. 7). When nitrite is supplied together with nitrate, a higher number of spatial jackpot events persist to the expansion edge at both pH 6.5 and 7.5, with the similar trend that a higher number of spatial jackpot events emerge at pH 6.5 than at 7.5 (Fig. 7a, c). The interaction strength is amplified at pH 6.5, where local detoxification of nitrite amplifies the growth difference between the two strains and results in more optimal growth conditions in close proximity to spatial jackpot events (Fig. 7a, c).

We further tested whether our results are robust to the initial redox conditions (Fig. 7a, b). When the fluctuations are initiated under oxic conditions, we observed higher numbers of spatial jackpot events persisting to the expansion edge at both pH 6.5 (mean = 7.75, SD = 1.25, *n* = 4) and 7.5 (mean = 3.5, SD = 1.29, *n* = 4). During the initial oxic phase, both the producer and consumer can proliferate, creating small pockets of kin cells. During the subsequent anoxic phase, the small pockets of kin cells have a higher chance of being shoved forward by the producer, and can thus form spatial jackpot events that protrude to the expansion edge. Therefore, regardless the initial redox condition, the strength of the interaction has a strong influence on the final spatial arrangement and number of spatial jackpot events.

## Discussion

In this study, we investigated how fluctuations in environmental conditions that alter interactions between two microbial strains influence the emergence and evolution of spatial self-organization. Using a microbial co-culture consisting of two strains that cross-feed nitrite (NO_2_^−^) under anoxic conditions and compete under oxic conditions, we conducted a series of range expansion experiments and complemented experimental observations with insights gained from a mechanistic agent-based model that mimics the experimental conditions. Overall, the emerging patterns of spatial self-organization are consistent with our previous observations of producer-first expansion under anoxic conditions and simultaneous expansion under oxic conditions [19] (Fig. 1b, c). They are also consistent with our expectation that repeated transitions between the two environmental conditions should result in increased abundance and dominance of the producer (Fig. 1d).

Contrary to our initial expectation (Fig. 1d), however, we found that the composition of the co-culture is preserved despite repeated transitions between anoxic and oxic conditions (Figs. 2–4). We attribute the stability in co-culture composition and spatial self-organization to the emergence of spatial jackpot events that enable the consumer to remain located at the expansion edge under anoxic conditions, and subsequently secure its position after transition to oxic conditions (Fig. 3). Thus, spatial jackpot events are an important mechanism that enables stable community composition in the face of environmental fluctuations and perturbations (Fig. 6). In essence, spatial jackpot events are a form of local spatial pattern diversity within microbial communities [56]. Thus, just as genetic diversity can provide compositional and functional stability to microbial communities [57–59], spatial pattern diversity can also contribute toward compositional and functional stability.

Why do spatial jackpot events emerge, and what enables their propagation? The term jackpot event has typically been used in relation to genotypic events, where rare mutations can emerge that enable new genotypes to proliferate and persist [37, 38, 60]. In our case, spatial jackpot events emerge from a stochastic process that does not have a genetic basis, as we demonstrated via heritability tests and genome re-sequencing analyses in a previous study [56]. Spatial patterns can diversify due to local variations in the initial spatial positionings of individual cells, which results in two different patterns of spatial self-organization that emerge simultaneously [44, 56]. The dominant pattern is “producer-first expansion”, where the producer expands first and the consumer follows. In this scenario, the expansion edge is occupied by producer cells that rapidly proliferate due to their preferential access to nitrate (NO_3_^−^) whereas initially negligible nitrite (NO_2_^−^) concentrations result in an exclusion of consumer cells from the expansion edge. The minority pattern is “consumer-first expansion” (referred to here as spatial jackpot events). During the development of a spatial jackpot event, the producer pushes a few consumer cells forward within the expansion area (Fig. 1b) [44, 56]. Our detailed modeling results show evidence for two important mechanisms that facilitate the nucleation of spatial jackpot events (Supplementary Fig. S6). First, the inoculated consumer cell needs to persist at the expansion edge via shoving by producer cells (Supplementary Fig. S6a). Furthermore, the results suggest that there is a stronger sensitivity to local conditions at pH 7.5, where the consumer benefits from a cluster of adjacent consumer cells supported by a background of producer cells in their vicinity that push the consumer cluster toward the expansion edge (Supplementary Fig. S6b, c). Once sufficient nitrite is available, the consumer cells that remain near the expansion edge gain a localized relative growth rate advantage due to abundant nitrite (in comparison to the diminishing nitrate availability per consumer cell) that results in the persistence of the observed spatial jackpot event (Fig. 5). In contrast, at pH 6.5 the local growth rate advantage of the consumer does not require a high number adjacent consumer cells in order to nucleate a spatial jackpot event (Supplementary Fig. S6b, c). Thus, stochastic processes determine the initial spatial positionings of individuals while deterministic processes then act on those individuals to generate a range of spatial patterns as a function of different relative growth rates and behaviors [33, 36, 61–63].

Previous studies that investigated range expansion in microbial communities highlighted that increasing the strength of a positive interaction can slow the loss of diversity under constant redox conditions [45]. We found that the persistence of the consumer is increased at the expansion edge in the face of environmental perturbations by strengthening the mutualistic interaction. The relative abundance of the two strains and also their intermixing showed comparable outcomes (Fig. 2), where the relative abundance and intermixing are both higher at pH 6.5 than at 7.5.

How generalizable are our main conclusions? Fluctuating environmental conditions frequently occur in natural systems such as in soils. Redox fluctuations following intermittent rainfall events, where anoxic conditions rapidly develop in saturated soils while oxic conditions prevail in unsaturated soils, expose soil microorganisms to fundamentally different environmental conditions that affect community composition and function [17]. Thus, our study may be of relevance for understanding the resistance and resilience of soil microbial communities to changes in redox. More generally, the principle that we investigated here may be relevant for any type of environmental perturbation or fluctuation conditional that the two assumptions discussed above are satisfied (i.e., different environmental conditions promote the emergence of different patterns of spatial self-organization and the patterns of spatial self-organization that emerge under one set of environmental conditions are detrimental under other sets of environmental conditions).

How widespread are spatial jackpot events likely to occur in nature? We argue that such spatial jackpot events may be typical features of self-organizing microbial communities. When any surface is colonized by microbial cells, individuals will not be distributed uniformly. Instead, colonized surfaces will contain local differences in the initial spatial positioning of individuals. These differences, in turn, can create spatial pattern diversity, where some of the patterns may provide new community-level properties such as resistance or resilience to environmental change. Thus, spatial jackpot events may be widespread and inevitable features of surface-associated microbial communities.

## Supplementary information

Supplementary Text

Supplementary Figure S1

Supplementary Figure S2

Supplementary Figure S3

Supplementary Figure S4

Supplementary Figure S5

Supplementary Figure S6

## Data Availability

All data and codes required to reproduce the figures and conclusions are publically available on the Eawag Research Data Institutional Collection (ERIC) repository at the following URL: https://data.eawag.ch/dataset/data-for-rare-and-localized-events.
