# Supplementary material for: Rare and localized events stabilize microbial community composition and patterns of spatial self-organization in a fluctuating environment
Source: ISME J. 2022 Jan 25;16(5):1453–63. doi: 10.1038/s41396-022-01189-9 (PMC9038690; doi:10.1038/s41396-022-01189-9)
Supplement: Supplementary file 1 — Supplementary Text [file 41396_2022_1189_MOESM1_ESM.pdf]

Supplementary Text

Rare and localized events stabilize microbial community composition and patterns of spatial self-organization in a fluctuating environment

Davide Ciccarese[1, 2, 3], Gabriele Micali [1, 2], Benedict Borer[2, 3], Chujin Ruan[1, 4], Dani Or[2, 5], David R. Johnson[1, *]

[1]Department of Environmental Microbiology, Swiss Federal Institute of Aquatic Science and Technology (Eawag), 8600 Dübendorf, Switzerland; [2]Department of Environmental Systems Science, ETH Zürich, 8092 Zürich, Switzerland; [3]Department of Earth, Atmospheric & Planetary Sciences, Massachusetts Institute of Technology, Cambridge, MA 02139, USA; [4]College of Land Science and Technology, China Agricultural University, 100193, Beijing, China; [5]Division of Hydrologic Sciences, Desert Research Institute, Reno, NE 89512, USA.

* - Corresponding author (david.johnson@eawag.ch)

This file includes:

Extended Materials and Methods

Supplementary Text References

Extended Materials and Methods

Range expansion experiments under continuous oxic or continuous anoic conditions. We performed control range expansion experiments with continuous oxic (n = 5) or continuous anoxic (n = 5) conditions using the same protocol and analytical approach as we described in the main text for the fluctuating experiment. The only exception was that we either maintained the LB agar plates in ambient air for the oxic controls or in the glove box containing a nitrogen (N_2):hydrogen (H_2) atmosphere (97:3) for the anoxic controls for the entire duration of the experiment.

Range expansion experiments with only the producer. We performed control range expansion experiments with only the producer (n = 5) using the same protocol and analytical approach as we described in the main text for mixtures of the producer and consumer. The only exception was that we did not add the consumer. The purpose of this control was to validate that the presence of the consumer improves the growth of the producer when nitrite (NO_2^-) is toxic, and thus to validate that the interaction is indeed mutualistic.

Residence time of oxygen in agar. To determine the residence time of oxygen in LB agar plates after transfer to our anoxic glove box, we inserted a microsensor oxygen needle (PreSens Precision Sensing GmbH, Regensburg, Germany) into an agar plate under oxic conditions. We then moved the agar plate into the glove box. We maintained the agar plate in the glove box until the oxygen concentration within the agar stabilized (Supplementary Fig. S2).

Image processing. We performed all image processing steps in ImageJ, Fiji 2.0.0 [1]. We first split the images into the different fluorescence channels. We next segmented the individual

fluorescence channels, recomposed the images, and identified the expansion edge. For the *ecfp* fluorescence channel images (producer images), we subtracted background noise with a rolling ball algorithm [2], enhanced the images with contrast-limited adaptive histogram equalization [3] followed by a gaussian filter, and binarized them with a default global threshold [4]. For the *egfp* fluorescence channel images, we used the same steps except we additionally segmented the images with a global threshold Renyi Entropy [5]. We then processed the *ecfp* and *egfp* segmented images similarly with a series of morphological operators (dilatation, filling and erosion operators) to fill small local gaps between the two channels. We next multiplied the resulting segmented images together and binarized them to obtain a mask of the expansion edge comprised of both genotypes. These images were then used to locate the expansion edge and measure the radius of the colony (Supplementary Fig. S3a, b).

To calculate the ratio of consumer-to-producer and the intermixing index, we used the same quantification approach described elsewhere [6, 7]. Briefly, we measured the ratio of consumer-to-producer and the intermixing index by sampling over a circumference superimposed on the binarized image (Supplementary Fig. S3c) located at the expansion edge with a width of 50 pixels (corresponding to a width of 151 µm). We counted the number of spatial jackpot events using an automated script in MATLAB R2017a (MathWorks, Nantick, MA, USA). First, we prepared the consumer-segmented images as described elsewhere [6, 7] via a sequential morphological operation with default settings as provided in ImageJ, Fiji 2.0.0 [1]. This returned consumer branches with closed edges and enabled us to count connected objects (Supplementary Fig. S3d). We evaluated the connected component process with the appropriate classification scores as described elsewhere [8–10]. Briefly, we compared the output with visual counting until obtaining a high concordance when calculating the ratio of correctly segmented image area over the ground

truth (accuracy; mean = 0.83, STD = 0.04, n = 5). Finally, we calculated the number of spatial jackpot events using connected component analysis at the expansion edge at the last time point (performed in Matlab R2017a) (Supplementary Fig. S3d).

Two-phase linear regressions. We fit the two-phase linear regression model to the ratio of consumer-to-producer and the intermixing index using the function *two_phase_lin* available in File Exchange on matworks.com [11].

SUPPLEMENTARY TEXT REFERENCES

1. Schindelin J, Arganda-Carreras I, Frise E, Kaynig V, Longair M, Pietzsch T, et al. Fiji: An open-source platform for biological-image analysis. Nat Methods. 2012;9:676-82.
2. Sternberg SR. Biomedical image processing. IEEE Comput. 1983;16:22-34.
3. Pizer SM, Johnston RE, Ericksen JP, Yankaskas BC, Muller KE. Contrast-limited adaptive histogram equalization: speed and effectiveness. In: Proceedings of the First Conference on Visualization in Biomedical Computing. 1990;337-45.
4. Ridler TW, Calvard S. Picture thresholding using an iterative selection method. IEEE Trans Syst Man Cybern. 1978;SMC-8:630-2.
5. Kapur JN, Sahoo PK, Wong AKC. A new method for gray-level picture thresholding using the entropy of the histogram. Comput Vision, Graph Image Process. 1985;29:273-85.
6. Goldschmidt F, Regoes RR, Johnson DR. Successive range expansion promotes diversity and accelerates evolution in spatially structured microbial populations. ISME J. 2017;11:2112-23.
7. Ciccarese D, Zuidema A, Merlo V, Johnson DR. Interaction-dependent effects of surface structure on microbial spatial self-organization. Philos Trans R Soc Lond B Biol Sci. 2020;375:20190246.
8. Thanh DNH, Sergey D, Surya Prasath VB, Hai NH. Blood vessels segmentation method for retinal fundus images based on adaptive principal curvature and image derivative operators. Int. Arch. Photogramm. Remote Sens. Spat. Inf. Sci. - ISPRS Arch. International Society for Photogrammetry and Remote Sensing, 2019;4212:211-8.
9. Thanh DNH, Erkan U, Surya Prasath VB, Kumar V, Hien NN. A skin lesion segmentation method for dermoscopic images based on adaptive thresholding with normalization of color models. Proc. - 2019 6th Int. Conf. Electr. Electron. Eng. ICEEE. 2019:116-20.

10. Thanh DNH, Prasath VBS, Hieu LM, Hien NN. Melanoma skin cancer detection method based on adaptive principal curvature, colour normalisation and feature extraction with the ABCD rule. J Digit Imaging. 2020;33:574-85.
11. Atanasov D. Two-phase linear regression model. MATLAB Central File Exchange. https://www.mathworks.com/matlabcentral/fileexchange/26804-two-phase-linear-regression-model.
