# Supplementary material for: Rare and localized events stabilize microbial community composition and patterns of spatial self-organization in a fluctuating environment
Source: ISME J. 2022 Jan 25;16(5):1453–63. doi: 10.1038/s41396-022-01189-9 (PMC9038690; doi:10.1038/s41396-022-01189-9)
Supplement: Supplementary file 2 — Supplementary Figure S1 [file 41396_2022_1189_MOESM2_ESM.pdf]

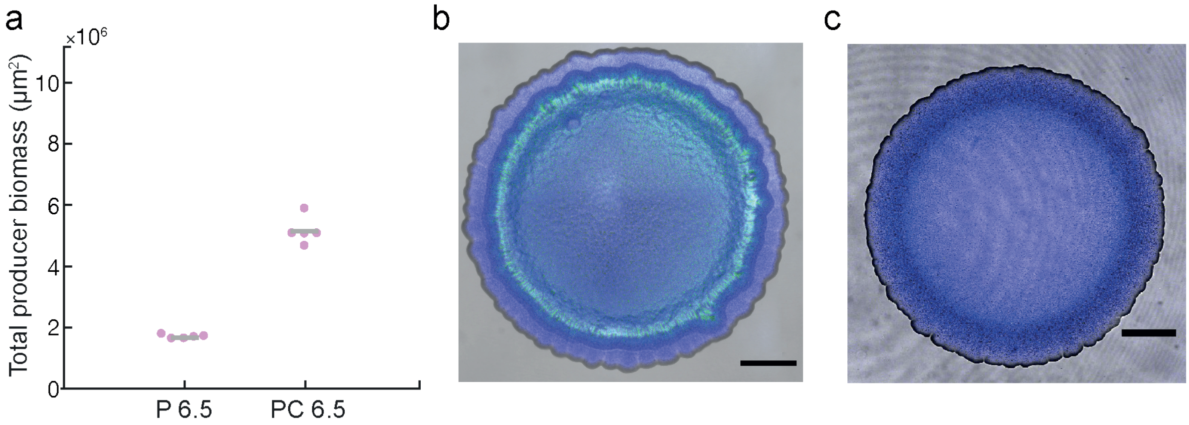

Supplementary Fig. S1: Effect of the consumer on growth of the producer during range expansion at pH 6.5 (strong mutualistic interaction). All range expansions were performed under anoxic conditions with nitrate (NO_3^-) as the growth-limiting nutrient. **a)** The presence of the consumer leads to a statistically significant increase in the growth of the producer. P, expansion of the producer alone; PC, expansion of the producer and consumer together. Each datapoint is for an independent replicate (n = 5). **b)** Representative image of the producer and consumer expanding together. The scale bar is 1000 μm. **c)** Representative image of the producer expanding alone. The scale bar is 1000 μm.
