# Supplementary material for: Rare and localized events stabilize microbial community composition and patterns of spatial self-organization in a fluctuating environment
Source: ISME J. 2022 Jan 25;16(5):1453–63. doi: 10.1038/s41396-022-01189-9 (PMC9038690; doi:10.1038/s41396-022-01189-9)
Supplement: Supplementary file 3 — Supplementary Figure S2 [file 41396_2022_1189_MOESM3_ESM.pdf]

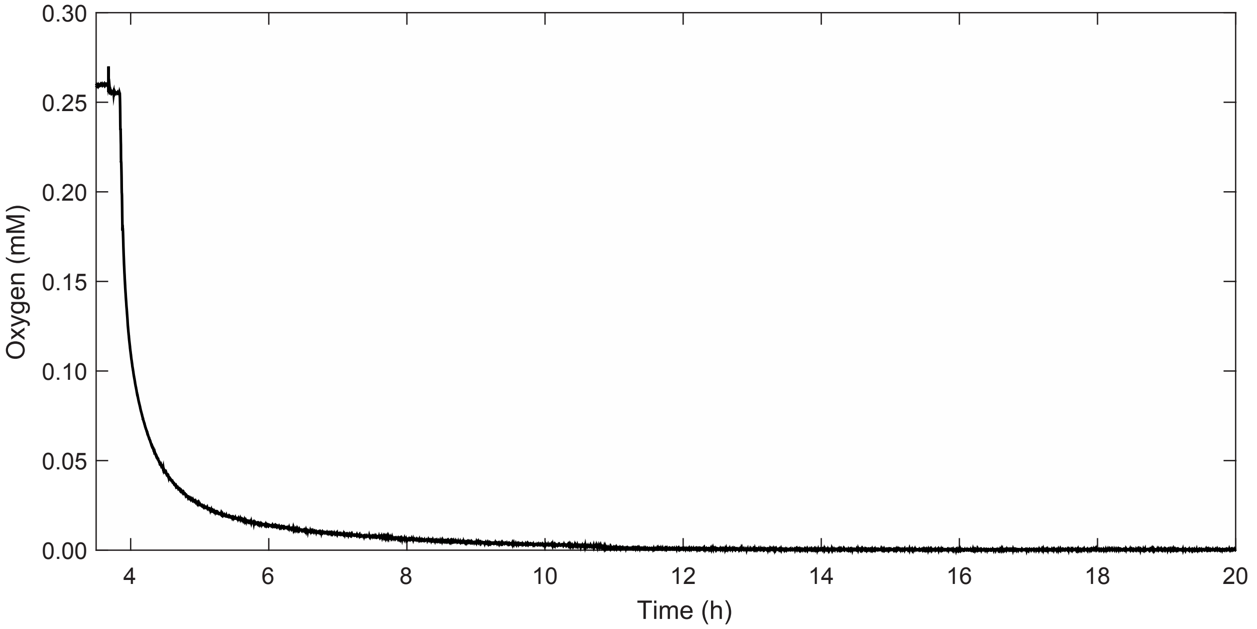

Supplementary Fig. S2: Agar oxygen concentration over time. LB agar plates were prepared in oxic conditions (ambient air) and then transferred into a glove box containing a nitrogen:hydrogen (97:3) anoxic atmosphere. Agar oxygen concentrations decreased rapidly and fell below 0.03 mM after approximately 5 h, at which point aerobic respiration is expected to cease.
