# Supplementary material for: Rare and localized events stabilize microbial community composition and patterns of spatial self-organization in a fluctuating environment
Source: ISME J. 2022 Jan 25;16(5):1453–63. doi: 10.1038/s41396-022-01189-9 (PMC9038690; doi:10.1038/s41396-022-01189-9)
Supplement: Supplementary file 4 — Supplementary Figure S3 [file 41396_2022_1189_MOESM4_ESM.pdf]

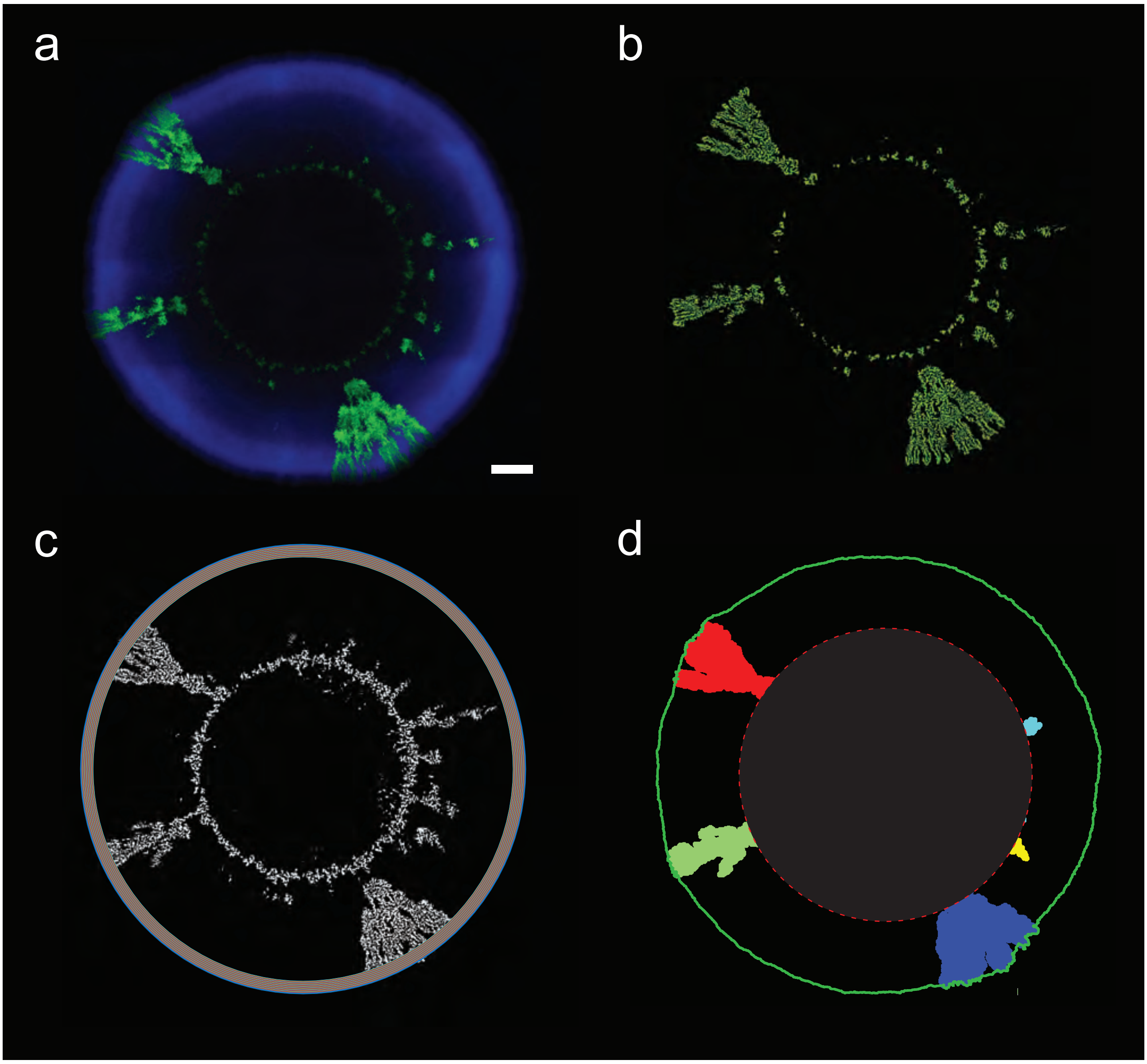

Supplementary Fig. S3: CLSM image processing of range expansions. a) CLSM image at the end of the anoxic-oxic fluctuation experiment (350 h). The producer is blue and the consumer is green. The scale bar is 1000 µm. **b)** Segmented image of the consumer. **c)** Radial sampling of the ratio of consumer-to-producer and the intermixing index. **d)** Connected component analysis used to count each spatial jackpot event. The red dashed line is a mask used to remove the inoculum area and determine the origin of each spatial jackpot event.
