# Supplementary material for: Rare and localized events stabilize microbial community composition and patterns of spatial self-organization in a fluctuating environment
Source: ISME J. 2022 Jan 25;16(5):1453–63. doi: 10.1038/s41396-022-01189-9 (PMC9038690; doi:10.1038/s41396-022-01189-9)
Supplement: Supplementary file 5 — Supplementary Figure S4 [file 41396_2022_1189_MOESM5_ESM.pdf]

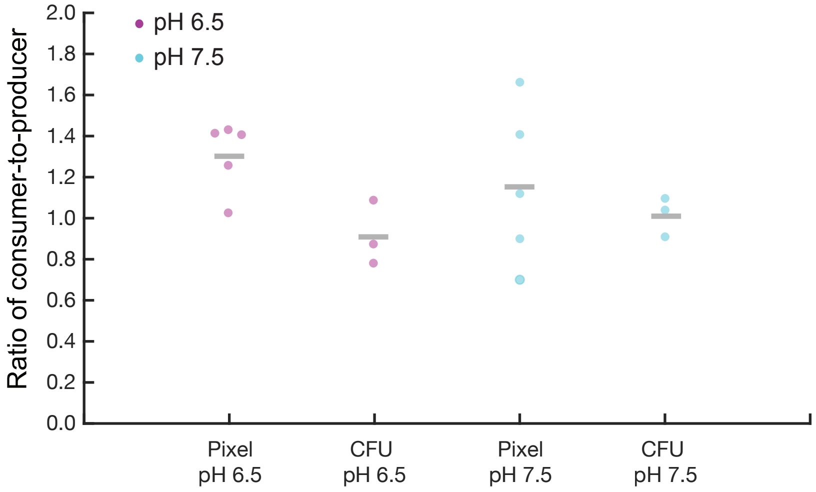


**Supplementary Fig. S4: Comparison of pixel- and colony forming unit (CFU)-based measures of the ratio of consumer-to-producer after range expansion.** All range expansions were performed under oxic conditions. The ratio of consumer-to-producer after 10 days of range expansion were measured across the entire expansion area using the ratio of green (consumer)-to-blue (producer) pixels or the ratio of green-to-blue CFUs. To count CFUs, the entire expansion area was dispersed in saline solution and a portion was then plated on LB agar plates containing IPTG to induce expression of the fluorescent proteins. Each data point is for an independent replicate (n = 5).
