# Supplementary material for: Rare and localized events stabilize microbial community composition and patterns of spatial self-organization in a fluctuating environment
Source: ISME J. 2022 Jan 25;16(5):1453–63. doi: 10.1038/s41396-022-01189-9 (PMC9038690; doi:10.1038/s41396-022-01189-9)
Supplement: Supplementary file 6 — Supplementary Figure S5 [file 41396_2022_1189_MOESM6_ESM.pdf]

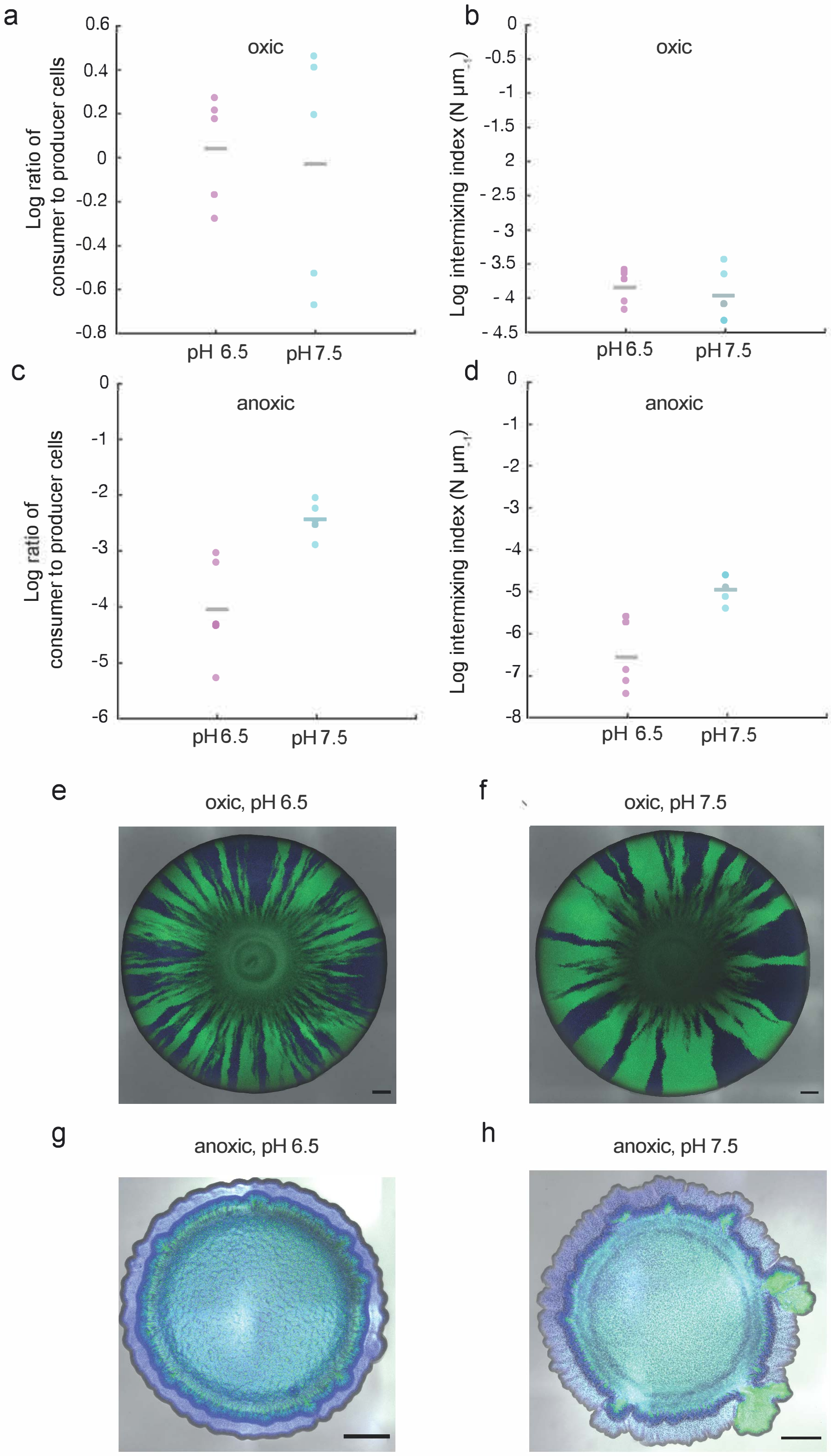

Supplementary Fig. S5: Analysis of range expansions under continuous oxic and continuous anoxic conditions. All oxic range expansions were performed in ambient air and all anoxic range expansions were performed with nitrate (NO_3^-) as the growth-limiting nutrient. Under continuous oxic conditions, there was no statistically significant difference in **a)** the ratio of consumer-to-producer, or **b)** the intermixing index between the two pH conditions. Under continuous anoxic conditions, **c)** the ratio of consumer-to-producer, and **d)** the intermixing index were both significantly larger at pH 7.5 than at 6.5. Each data point is for an independent replicate (n = 5) Representative images at the end of the range expansions for **e)** oxic conditions at pH 6.5, **f)** oxic conditions at pH 7.5, **g)** anoxic conditions at pH 6.5, and **h)** anoxic conditions at pH 7.5. All scale bars are 1000 μm.
