# Supplementary material for: Rare and localized events stabilize microbial community composition and patterns of spatial self-organization in a fluctuating environment
Source: ISME J. 2022 Jan 25;16(5):1453–63. doi: 10.1038/s41396-022-01189-9 (PMC9038690; doi:10.1038/s41396-022-01189-9)
Supplement: Supplementary file 7 — Supplementary Figure S6 [file 41396_2022_1189_MOESM7_ESM.pdf]

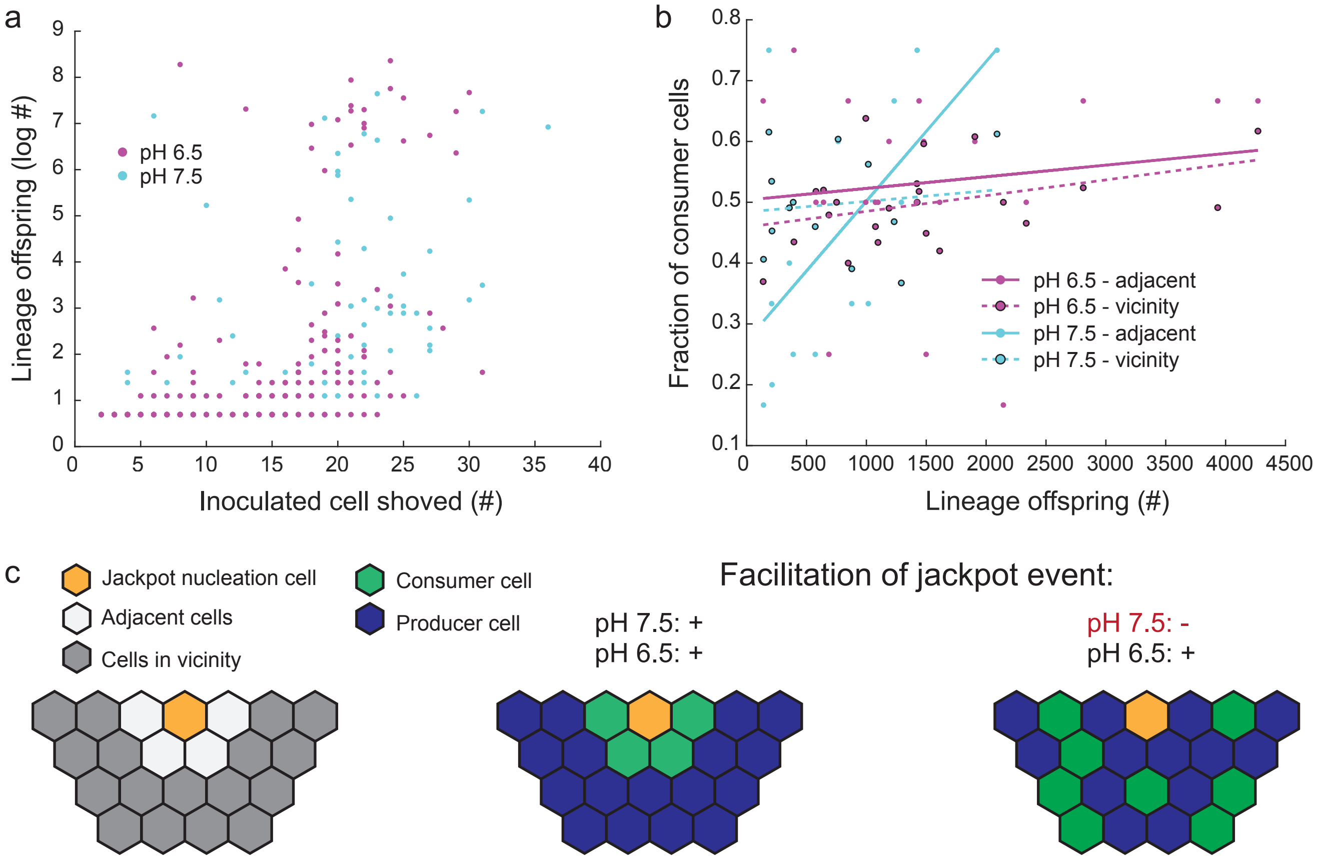

Supplementary Fig. S6: Mechanistic processes facilitating the emergence of spatial jackpot events. a) Relationship between the number of times an inoculated consumer cell was shoved and the resulting proliferation of the lineage (counted as number of offspring). More pronounced shoving of the consumer cells by the producer generally results in a higher probability of lineage proliferation (in the form of a spatial jackpot event). **b)** Relationship with linear regression fits between the lineage offspring and fraction of consumer cells around the inoculated consumer cell for the direct adjacent cells and cells in close vicinity. At pH 6.5, there is no significant relationship between the spatial structure of the neighboring cells. At pH 7.5, consumer cells benefit of residing in small clusters (other consumer cells directly adjacent) that is surrounded by a more balanced ratio of consumers and producers in the local vicinity. **c)** Explanation of the spatial jackpot nucleating cell, adjacent cells and cells in close vicinity including two scenarios that facilitate the emergence of spatial jackpot events as a function of pH.
